# The prevalence of and factors associated with tobacco smoking behavior among long-distance drivers in Lagos, Nigeria

**DOI:** 10.4314/ahs.v17i3.32

**Published:** 2017-09

**Authors:** Obianuju B Ozoh, Maxwell O Akanbi, Casmir E Amadi, William Vollmer, Nigel Bruce

**Affiliations:** 1 University of Lagos College of Medicine; Lagos University Teaching Hospital, Department of Medicine; 2 Jos University Teaching Hospital, Internal Medicine; Northwestern University, Center for Global Health; 3 Kaiser Permanente Center for Health Research, Portland, Oregon, United States of America; 4 University of Liverpool, United Kingdom, Department of Public health and Policy

**Keywords:** Tobacco smoking behavior, long-distance drivers, Lagos, Nigeria

## Abstract

**Background:**

Factors associated with tobacco smoking are useful in designing tobacco control programs.

**Objectives:**

To estimate the prevalence of and factors associated with tobacco smoking among long-distance drivers.

**Methods:**

This was a cross-sectional study. Stratified cluster sampling approach was used to select drivers based on if they received annual health screening (AHS) or not (non AHS). We used a structured questionnaire to obtain information and weighted the resulting observations to derive population based estimates. Association between tobacco smoking and socio-demographic factors was explored in multivariate models.

**Results:**

414 male drivers, mean age 43.6 (standard error 0.6) years. Population weighted prevalence of current smoking was 18.9% (95% CI: 14.3–23.4) of all drivers, 6.5% (95% CI: 2.6–10.4) of AHS drivers and 19.5 (95% CI: 14.7–24.2) of non AHS drivers (p<0.001). In multivariate models, having close friends that smoked (OR= 6.36, 95% CI= 2.49 – 16.20) cargo driving (OR= 2.58, 95% CI= 1.29 – 5.15) and lower education levels (OR for post-secondary education vs. primary education or less= 0.17, 95% CI= 0.04 – 0.81) were associated with current smoking.

**Conclusion:**

Prevalence of tobacco smoking is higher among non AHS compared to AHS drivers. Having close friends that smoked, cargo driving, and lower education levels were associated with current smoking.

## Introduction

Tobacco use is a public health problem, with the worldwide tobacco-attributable deaths projected to be 8.3 million in 2030^1^. It also poses a substantial economic burden on the individuals who consume it and on the health care system^2^.

Despite a relatively low prevalence of cigarette smoking and tobacco use in the general population in Nigeria (6%), commercial drivers have a high prevalence of cigarette smoking (25–85%)^3^–^7^. Previous studies have suggested that high cigarette smoking rates among long distance commercial drivers is related to the high stress level associated with the job and to peer pressure^4^. Other factors, such as socio-demographic status, family and societal values, knowledge of harmful health effects of tobacco use, and work place screening programs, have not been extensively evaluated among this occupational group^8^.

In 2014, the Lagos State government enacted a tobacco control law that banned smoking in public places and the sale of tobacco to and by minors^9^. It also imposed a comprehensive ban on advertising, sponsoring, and promoting tobacco products and required that cigarette packets contain graphic health warnings highlighting the harmful health effects of tobacco smoking. The awareness and understanding of this new law among commercial drivers has not been evaluated.

We conducted a cross-sectional study first to estimate cigarette smoking prevalence among commercial long-distance drivers operating from Lagos, Nigeria and also to evaluate the association between having access to annual health screening as well as other socio-demographic factors and their use of tobacco. We also aimed to determine the awareness and understanding of the new tobacco control law among long-distance drivers operating from Lagos, Nigeria.

## Methods

This was a cross-sectional study. The Health Research Ethics Committee of the Lagos University Teaching Hospital, Lagos, Nigeria approved all study procedures. We obtained permission and endorsement for the study from the heads of motor parks and transport companies and informed consent from all participants.

## Participant selection and recruitment

**Figure 1:**
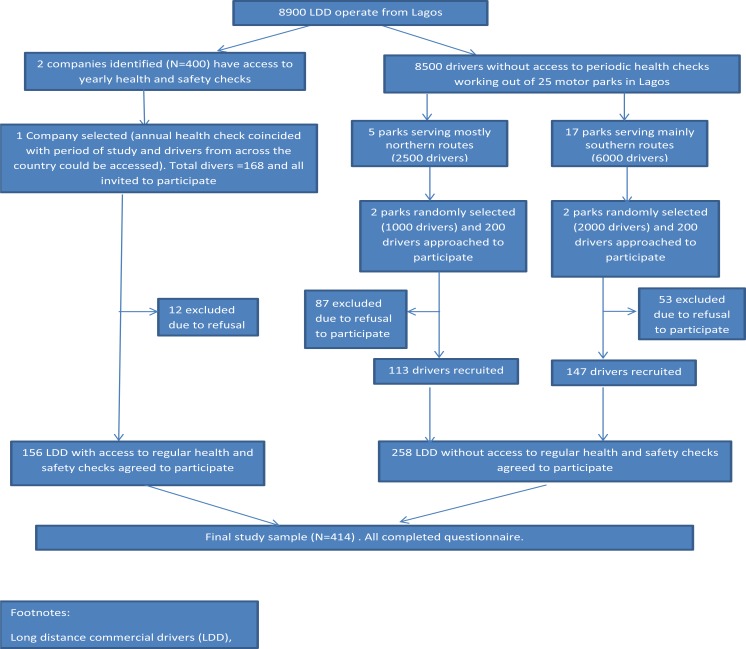
Consort diagram describing how participants were recruited into the study

Lagos metropolis is the commercial capital of Nigeria, with a growing population of over 17 million people. There is a high rate of movement of goods, services and commuters between Lagos and all parts of the country, often through road networks by commercial drivers. We used a stratified cluster sampling approach to recruit long-distance drivers registered with the National Union of Road Transport Workers (NURTW) from selected motor parks across Lagos between March and July 2015 (figure 1). We first stratified motor parks based on whether or not their drivers were registered and they organized mandatory formal annual health and safety training and assessment for their drivers (AHS motor parks). Only two motor parks (together employing about 400 drivers) met these criteria. The drivers in the AHS motor parks only operate from their company terminals and ply routes across Nigeria. We selected one of these two motor parks for inclusion in the study. It was chosen because its annual health and safety program coincided with the timeframe during which our study was conducted. All 168 drivers at this motor park were invited to participate, though 12 declined (92.9% agreeing to participate).

The second category of (non AHS) motor parks were composed of independent drivers and drivers working for small transport companies that operate from general motor parks in Lagos, Nigeria. The drivers in these motor parks are generally less regulated and do not routinely receive formal health and safety checks. We divided these motor parks into those primarily serving the Northern part of the country and those primarily serving the Southern part of the country. We then randomly selected two motor parks from each of these strata for inclusion in the study, thereby selecting four in total. Finally, we approached a convenience sample of 100 drivers from each of these four parks and ultimately recruited 258 of them (64.5% agreeing to participate). Those who declined mainly did so due to time constraints.

## Data collection

We used a structured questionnaire administered by trained interviewers to obtain socio-demographic data; self-reported use of tobacco products, alcohol, other psychoactive substances; knowledge of the harmful effects of tobacco smoking; and awareness and understanding of the tobacco control law. The questionnaire took about 15 to 20 minutes to complete.

Based on standard definitions, a cigarette smoker (ever smoker) was a person who had smoked at least 100 cigarettes in their lifetime or who had smoked at least one cigarette per day for one year, and a current smoker as an ever smoker who smoked at least once in the preceding week. Smoking intensity was reported as pack years which was calculated as the average number of cigarettes smoked per day divided by 20 and multiplied by the number of years of smoking^10^.

For current smokers, we assessed the level of tobacco dependence using the Fagerström Test for nicotine dependence. This is a validated 6 item questionnaire that asks about time of first cigarette in a day, smoking in forbidden places, most difficult cigarette to give up, number of sticks of cigarette smoked per day, smoking the highest quantity of cigarette in the first hour after waking and smoking when quite ill. Each question has options that are weighted one to three with a maximum total score of ten. A total score of 0–4 shows mild nicotine dependence, 5–6 medium dependence and 7–10 high nicotine dependence^11^. We also administered the Smokers Emotional index questionnaire (SEI) which provides a measure of smokers' emotional balance and has been hypothesized to correlate with the probability of successful quitting. The index ranges from 0 to 18 with higher score indicating worse emotional status^12^.

Regular alcohol consumption was defined as taking an equivalent of at least 8 units of alcohol (one drink of an alcoholic beverage) 3 or more times a week. Kolanut is a local fruit with very high caffeine content, and regular use was regarded as eating kolanuts at least 4 times a week. The questionnaire was translated and back translated into Pidgin English and we used either the original English or translated version depending on the drivers' preference.

## Statistical methods

Because drivers from the AHS motor parks are disproportionately represented in our sample, we calculated sampling weights for each study participant and used these to calculate valid population-based estimates for the cohort as a whole. Accordingly the AHS drivers contribute very little to the overall population estimates.

We conducted all analyses in Stata, version 12.1, using the survey statement to provide suitably weighted population-based estimates of all means, prevalences, and regression coefficients. We used Wald tests from unadjusted linear regression models to compare means and proportions between groups for and for multivariate logistic regression models to evaluate the significance of individual variables. When used with the survey statement, the Wald statistics from linear and logistic regression models are treated as t-statistics or, for polychotomous variables with 3 or more levels, F statistics.

The study was powered to provide a margin of error of approximately ±5 percentage points for estimating the prevalence of smoking in the population overall (ignoring sampling) assuming a 95% confidence interval, an estimated smoking prevalence of 25%, and a sample size of 289 drivers.

Unless otherwise stated, the term statistically significant refers to a two-sided p-value ≤0.05.

## Results

Table 1 describes the population characteristics of long distance drivers both overall and by AHS status. The mean age of the drivers was 44 years and ranged from 22 years to 76 years. The mean duration of driving was 19 years and ranged from 2 to 55 years. Non AHS drivers were significantly less educated (p<0.001), but earned significantly more (p<0.001) than the AHS drivers. All cargo drivers were non-AHS drivers (p<0.001). Despite a similar awareness of the tobacco control law, more AHS drivers had a good understanding of the law (p=0.009). Knowledge of harmful health effects of smoking was modest for most conditions (65%–75%), except for respiratory illnesses and infertility (17%–38%) and generally did not differ significantly between the groups. Only one third of the drivers knew that cigarette smoking affects others who do not smoke. Perception of a negative community attitude towards tobacco smoking did not differ significantly between the groups but perception of a negative religious attitude towards smoking was significantly higher among AHS drivers (p<0.001).

**Table 1:** Estimated population characteristics of long distance drivers in Lagos.^1^

Characteristics	Overall Population (weighted) (414)	Drivers with access to health screening (156)	Drivers without access to health screening (258)	P value
**Age in years, mean (SE)**	43.7 (0.6)	43.8 (0.7)	43.7 (0.7)	0.93
**Duration of driving in years,** **mean (SE)**	18.9 (0.7)	18.8 (0.8)	18.9 (0.7)	0.91
**Highest level of education (%)**				
**Primary or less**	34.3	6.5	35.5	<0.001
**Secondary**	59.5	53.2	59.8	
**Post-secondary**	6.2	40.3	4.6	
**Cargo driver (%)**	28	0	29.3	<0.001
**Married (%)**	92.7	90.9	92.8	0.51
**Monthly income in thousands of Naira (%)**				
**<30**	1.9	10.4	1.5	<0.001
**30–80**	73	79.2	72.7	
**>80**	25.1	10.4	25.8	
**Religion is Islam (%)**	27.6	1.3	28.8	<0.001
**Aware of tobacco control law** **(%)**	81.1	74.7	81.4	0.12
**Good understanding of** **tobacco control law (%)**	43.6	56.5	43	0.009
**Perception of a negative** **community attitude towards** **cigarette smoking (%)**	58.6	56.5	58.7	0.67
**Perception of a negative** **religious attitude towards** **cigarette smoking (%)**	79	96.1	78.2	<0.001
**Perceived effects of smoking**				
**Heart attack (%)**	66.5	63.6	66.6	0.55
**Lung cancer (%)**	68.9	76	68.6	0.11
**Bronchitis (%)**	37.7	33.1	38	0.33
**COPD (%)**	17.8	16.9	17.8	0.81
**Yellow teeth (%)**	71.6	74.7	71.5	0.48
**Bad breath (%)**	73.4	76	73.3	0.55
**Wrinkles (%)**	44	30.5	44.7	0.004
**Infertility (%)**	29.2	37	28.8	0.1
**Affects others (%)**	37.5	39.6	37.4	0.66
**Any of above (%)**	66.9	65.6	66.9	0.78

## Prevalence and pattern of cigarette smoking

Table 2 shows the prevalence of cigarette smoking and use of other psych-oactive substances among the drivers. Although the prevalence of ever smoking did not differ significantly between AHS drivers and non-AHS drivers, the prevalence of current smoking was significantly lower among AHS drivers compared to non-AHS drivers (6.5% versus 19.5%, p<0.001). The mean scores on the Fagerström Test, while significantly different between AHS (1.2) and non AHS (2.1) drivers (p=0.03) were consistent with mild nicotine dependence. Mean SEI scores did not differ significantly between the groups and were in a range that suggested that the emotional status of the smokers on the average was good. Over eighty percent of all current smokers desired to quit smoking.

**Table 2:** Estimated population prevalence and pattern of cigarette smoking and psycho-active substance use among long distance drivers in Lagos.^1^

	Overall Population (weighted) (N=414)	Drivers with access to health screening (N=156)	Drivers without access to health screening (N=258)	P value
**Cigarette smoking**				
Prevalence of ever smoking, % (CI)	25.5 (20.5–30.6)	22.7 (16.0–29.4)	25.7 (20.4–30.9)	0.50
Prevalence of current smoking, % (CI)	18.9 (14.3–23.4)	6.5 (2.6–10.4)	19.5 (14.7–24.2)	<0.001
Age at onset for all ever smokers, mean (SE)	21.9 (0.9)	24.8 (1.5)	21.8 (1.0)	0.09
Pack years for current smokers, mean (SE)	7.6 (1.2)	3.0 (0.7)	7.6 (1.2)	0.001
Fagestrom's test score for current smokers, mean (SE)	2.1 (0.3)	1.2 (0.3)		
SEI score for current smokers, mean (SE)	2.8 (0.3)	4.3 (0.8)	2.7 (0.3)	0.08
Current smokers who desire to quit % (CI)	82.4 (71.5–93.4)	90.0 (67.4–100)	82.3 (71.2–93.4)	0.50
**Cannabis use**				
Ever smokers, % (CI)	15.3 (10.9 – 19.7)	6.5 (2.6 – 10.4)	15.7 (11.1 – 20.4	0.003
Current smokers, % (CI)	10.8 (6.9 – 14.7)	6.5 (2.6 – 10.4)	11.0 (6.9 – 15.0)	0.12
Number of wraps per day, mean (SE)	5.0 (1.0)	3.0 (0.4)	5.0 (1.0)	0.07
**Current Kolanut use, % (CI)**	34.6 (29.0 – 40.1)	46.1 (38.1 – 54.1)	34.0 ( 28.2 – 39.8)	0.02
**Current smokeless tobacco use,** **% (CI)**	0.6 (0 – 1.2)	1.3 (0 – 3.1)	0.5 (0 – 1.2)	0.43
**Current alcohol drinking, %** **(CI)**	56.3 (50.2 – 62.3)	59.7 (51.9 – 67.6)	56.1 (49.8 – 62.4)	0.48

1 Estimates constructed using sampling weights and are meant to reflect characteristics of the universe of motor park drivers operating out of Lagos overall and in each subgroup. SE= Standard error of mean, CI=95% confidence interval, SEI=Smokers' Emotional Index

## Factors associated with current cigarette smoking

Compared to non-smokers, current smokers were more likely to be cargo drivers (p=0.001), have friends that smoke cigarettes (p<0.0010, and smoke cannabis (p=0.02) as shown in Table 3.

**Table 3:** Estimated population characteristics of current smokers andnon-smokers.^1^

Characteristics	Cigarette smokers	Non-smokers	P value
**Mean age (SE)**	42.4 (1.3)	44.0 (0.7)	0.32
**Mean duration of driving (SE)**	17.4 (1.3)	19.3 (0.8)	0.23
**Awareness of tobacco law % (CI)**	84.2 (75.6 – 92.9)	80.4 (75.1 – 85.7)	0.46
**Good understanding of tobacco** **law % (CI)**	46.0 (32.5 – 59.6)	43.0 (36.3 – 49.7)	0.69
**Type of driver (Cargo)**	47. 4 (35.6 – 59.2)	23.5 (20.4 – 26.6)	0.001
**Had a family member that smoked** **cigarettes while growing up % (CI)**	46.3 (32.4 – 60.1)	42.4 (35.6 – 49.1)	0.61
**Has close friend that smoke** **cigarettes %(CI)**	89.9 (81.5 – 98.2)	57.2 (50.5 – 63.9)	<0.001
**Currently smoke cannabis %(CI)**	22.8 (10.9 – 34.7)	8.0 (4.2 – 11.8)	0.02
**Currently drinking alcohol** **regularly %(CI)**	66.9 (53.7 – 80.0)	53.8 (47.0 – 60.6)	0.08
**Currently using kolanuts % (CI)**	43.7 (30.3 – 57.0)	33.7 (27.5 – 39.9)	0.18

1 Estimates constructed using sampling weights and are meant to reflect characteristics of the universe of motor park drivers operating out of Lagos overall and in each subgroup. CI=95% confidence interval.

In multiple logistic regression analysis (Table 4), having close friends that smoked (odds ratio (OR) = 6.36, 95% CI= 2.49 – 16.20) and being a cargo driver (OR= 2.58, 95% CI= 1.29 – 5.15) were significantly associated with being a current smoker, while being more educated (post-secondary education specifically), reduced the odds of being a current smoker (OR for post-secondary education vs. primary education or less =0.17, 95% CI= 0.04 – 0.81). Note that, because none of the drivers in AHS parks were cargo drivers, estimation of the effect of AHS in Table 4 is effectively limited to non-cargo drivers, while estimation of the effect of cargo driving is limited to drivers from non-AHS parks.

**Table 4:** Multivariate Logistic regression analysis for factors associated with current smoking^1^

Factors	Adjusted odds ratio	95% CI	P value
**Age**	0.92*	0.56 – 1.50	0.73
**Years as a driver**	0.84*	0.56 – 1.27	0.41
**Had a family member who smoked** **cigarettes while growing up**	0.98	0.50 – 1.94	0.96
**Has close friends who smoke** **cigarettes**	6.36	2.49 – 16.20	<0.001
**Type of park (AHS)**^2^	0.78	0.33 – 1.86	0.57
**Being a cargo driver**^3^	2.58	1.29 – 5.15	0.007
**Level of education**			
**Primary education**	Reference		
**Secondary education**	0.75	0.35 – 1.59	0.45
**Post-secondary education**	0.17	0.04 – 0.81	0.03
**Married**	2.67	0.72 – 9.82	0.14
**Aware of tobacco law**	1.53	0.61 – 3.83	0.37
**Understand tobacco law**	1.18	0.56 – 2.50	0.66
**Receiving talk on danger of cigarette** **smoking**	1.09	0.54 – 2.22	0.8

1 Individual observation weighted according to sampling fractions.

2 Effect of AHS versus non AHS among non-cargo drivers

3 Effect of cargo versus non-cargo among drivers in non AHS parks

CI= Confidence interval.

* Odds ratio for 10 year increase in age and duration of driving, respectively.

## Discussion

The overall prevalence of cigarette smoking among long-distance drivers in Lagos, Nigeria is high compared to reported prevalence in the general population. However, when considered by AHS status, the prevalence among AHS drivers is similar to that in the general population. Although the AHS drivers had significantly lower likelihood of being current smokers, this benefit was no longer significant after adjusting for confounders. Having close friends who smoke, cargo driving, and lower education levels were independently associated with higher odds of current smoking.

Another important finding from this study is a high rate of use of other psycho-active substances among long distance drivers compared to the general Nigerian population and the significant association between current smoking and the use of cannabis and alcohol. A recent national survey of substance use in the Nigerian general population reported the current use of cannabis and alcohol as 2% and 25% respectively which is much lower than reported in our study and this suggests that peculiar factors are likely to drive social habits among commercial drivers^13^. For example in this study despite the recognition by the majority of drivers that there was a negative religious and community perception towards cigarette smoking, a high proportion of them still smoke cigarettes. One of the major strengths in our study is the use of improved methodology for participant selection and statistical analysis. We calculated weighted population based estimates of means, prevalences and regression coefficients. This implies that our results are more likely to be generalizable to the population of long distance drivers across the country. Another strength in this study is that we evaluated the association between regular health screening among drivers and the prevalence of cigarette smoking in Nigeria because it brings to the fore an important social distinction among long distance drivers and the potential association it may have with smoking behavior. A recognized limitation in this study is that the prevalence of cigarette smoking was based on self-report which may be unreliable and biomarkers of recent smoking such as urinary or salivary cotinine was not obtained for validation^7^. However, self-report is widely used for estimating population prevalence of tobacco use in most international surveys and is generally accepted to provide a reasonable estimate^3^.

The prevalence of current smoking in this study corroborates previous reports from Nigeria as well as other parts of the world regarding the high rates of cigarette smoking among long distance drivers relative to the general population^4^,^14^–^18^. The figures obtained in this study for current smoking however are lower than has been reported in previous studies among long distance drivers in Nigeria (26%–44%)^14^–^16^. Most previous studies were conducted in general motor parks among non-AHS drivers who are generally less educated and as reported in this study primary education and below may be associated with a higher likelihood of smoking^19^,^20^.

Peer pressure is a recognized driving force for cigarette smoking and in our study having friends who smoke which may lead to peer pressure also increased the odds of being a current smoker^21^–^23^. However, we cannot conclude from this cross-sectional study that this association implies causality, although it is likely, but it is reasonable to assume that smoking partly drives social networks and maybe vice versa. Curiously, family smoking, which has been found to be an important factor associated with current smoking in other studies^22^,^23^, was not an important correlate of current smoking in this study. Probably, exposures in adult life such as peer pressure and availability of cigarettes may impact on current smoking status more than early life experiences.

Although all cargo drivers in this study were non-AHS drivers and raises the potential for confounding, we adjusted accordingly by including both indicators of AHS parks and cargo driving in the multivariate model. Even then, interpretation of the resulting coeffients is somewhat constrained. However, this association between current smoking and being a cargo driver is plausible; unlike passenger drivers who are usually not permitted by passengers to smoke while driving, cargo drivers can smoke without restraint. Previous studies among petroleum tanker drivers have also reported very high rates of cigarette smoking. For example 50% of petroleum product tanker drivers in Nigeria smoked cigarettes^26^. Cargo drivers therefore should be recognized as a sub-group of commercial drivers to target in the implementation of effective tobacco control programs.

Regarding the use of other psycho-active substances, our finding corroborates previous reports of high rates of psycho-active substance use among long distance drivers. Most drivers refer to fighting fatigue and promoting alertness as the reason for use; however, psycho-active substances have been associated with increased risk of road traffic accidents (RTAs)^4^,^14^,^21^,^27^–^28^. Although a study in Australia has reported reduced RTAs in users of caffeinated products, it is important to note that the Australian drivers are usually regulated and have a maximum number of driving hours stipulated by law which is not the case for most long distance drivers in Nigeria^29^.

## Conclusion

The estimated population based prevalence of cigarette smoking is high among long distance drivers operating from Lagos, Nigeria. Drivers who participated in annual health screening had significantly lower likelihood of being current smokers, although this benefit was no longer significant after adjusting for confounders. It is unclear to what extent the lower smoking prevalence seen in AHS drivers was due to the health screenings and education they receive as opposed to other factors that distinguish this group from the non AHS drivers. For instance none of the AHS drivers was a cargo driver. That does not necessarily mean that the health education and screenings did not matter, rather, they may have been influenced by some of the other factors adjusted for in the multivariate model. In multivariate models, having close friends who smoke, cargo driving, and lower education levels were independently associated with higher odds of current smoking. This study highlights that long distance drivers are an important target group for tobacco control interventions. Strategies to provide regular health screening may provide additional benefits by influencing smoking behavior and is worth exploring in future studies.
